# Clostridium difficile Infection Is Associated With Decreased Prostate Cancer Risk: A Retrospective Cohort Study

**DOI:** 10.7759/cureus.34398

**Published:** 2023-01-30

**Authors:** Lexi R Frankel, Amalia Ardeljan, Nadia G Obaed, Kazuaki Takabe, Omar Rashid

**Affiliations:** 1 Dr. Kiran C. Patel College of Allopathic Medicine, Nova Southeastern University, Fort Lauderdale, USA; 2 Michael and Dianne Bienes Comprehensive Cancer Center, Holy Cross Health, Fort Lauderdale, USA; 3 Department of Surgical Oncology, Roswell Park Comprehensive Cancer Center, Buffalo, USA; 4 Department of Surgery, The State University of New York, Buffalo, USA; 5 Leonard M. Miller School of Medicine, University of Miami, Miami, USA; 6 Department of Surgical Oncology, Massachusetts General Hospital, Boston, USA; 7 Department of Surgical Oncology, Broward Health, Fort Lauderdale, USA; 8 Department of Surgical Oncology, TopLine MD Alliance, Fort Lauderdale, USA; 9 Department of Surgical Oncology, Memorial Health, Pembroke Pines, USA; 10 Department of Surgical Oncology, Delray Medical Center, Delray, USA

**Keywords:** clostridium difficile infection, clostridium difficile treatment, prostate cancer, cytokines, interleukins, cancer prevention

## Abstract

Background

*Clostridium difficile* (*C. difficile*) is one of the most common hospital-acquired infections and causes the release of various cytokines. Prostate cancer (PC) is the second most common cancer in men worldwide. As infections have been associated with decreased cancer risk, the effects of *C. difficile* on the risk of developing PC were analyzed.

Methodology

Using the PearlDiver national database, a retrospective cohort analysis was performed to evaluate the relationship between a prior history of *C. difficile *infection and subsequent development of PC. International Classification of Disease Ninth and Tenth Revision codes were used to evaluate the incidence of PC between January 2010 and December 2019 in patients with and without a history of *C. difficile* infection. The groups were matched by age range, Charlson Comorbidity Index (CCI), and antibiotic treatment exposure. Standard statistical methods, including relative risk and odds ratio (OR) analyses, were utilized to test for significance. Demographic information was subsequently analyzed and compared between experimental and control groups.

Results

A total of 79,226 patients were identified in both the infected and control groups matched by age and CCI. The incidence of PC was 1,827 (2.56%) in the *C. difficile* group and 5,565 (7.79%) in the control group (p < 2.2 × 10^-16^; OR = 0.390, 95% confidence interval (CI) = 0.372-0.409). Subsequent matching by antibiotic treatment resulted in two groups of 16,772 patients. PC incidence was 272 (1.62%) in the *C. difficile* group and 663 (3.95%) in the control group (p < 2.2 × 10^-16^; OR = 0.467, 95% CI = 0.431-0.507).

Conclusions

Results from this retrospective cohort study demonstrate that *C. difficile* infection is associated with a reduced incidence of PC. Future studies are recommended to investigate the potential effect of the immune system and cytokines related to *C. difficile* infection on PC.

## Introduction

Prostate cancer (PC) is the second most common and fifth most deadly cancer in men worldwide, and its incidence is increasing [1]. While most forms of PC are slow-growing, one-third of patients develop more aggressive forms that can quickly lead to metastasis and death [2]. Worldwide, there are 300,000 PC deaths and over 1,100,000 new cases each year [2].

Several factors have been associated with an increased risk of PC, including a family history of PC, older age, African American race [3], and high consumption of alpha-linolenic acid [4]. In contrast, there have been much fewer identified factors associated with a decreased risk of PC. Possible preventative factors include increased physical activity and increased consumption of cruciferous vegetables and tomato sauce [3,4]. Although our knowledge of risk and protective factors has grown in recent years, there are still no concrete recommendations for preventative interventions for PC, and further protective factors for PC need to be identified [4].

While immunotherapy has demonstrated success in various cancers, its success in PC has been controversial. Some studies have reported the promise of immunotherapy for PC, while others have fervently reported its lack of success, calling it “immunologically cold” [2]. Within the past 10 years, there have been several clinical trials assessing programmed death receptor 1 (PD-1) and cytotoxic T-lymphocyte antigen-4 (CTLA-4) inhibitors for the treatment of metastatic castration-resistant PC [2,5]. The results of these trials have shown very limited benefits [5]. However, there have been more promising studies on vaccines and immune checkpoint therapies [5].

More recently, studies have referenced the importance of focusing on targeting cytokine-containing immune pathways, immune intensification and immune modulation, the role of the microbiome in immunotherapy response, and identifying new biomarkers for PC development [6,7]. One potential pathogen that may play a surprising role in identifying such immune pathways and biomarkers for PC is *Clostridium difficile* (*C. difficile*). *C. difficile* is one of the most common hospital-acquired infections and causes a vigorous immune response. Infection with *C. difficile* causes the release of various proinflammatory cytokines, including interleukin (IL)-1β, IL-8, IL-16, and IL-17A, and several other regulatory and anti-inflammatory cytokines, including IL-10, IL-23, and IL-48 [8]. Some of these cytokines have been noted to be increased in states of inflammation and cancer; whether their presence stimulates carcinogenesis or if cancer stimulates tumoricidal cytokine recruitment has been debated profusely [9]. If the latter is true, then the cytokines released by *C. difficile *infection may prove useful in reducing PC development. Vigorous infections and vaccination-induced immune responses have previously been associated with decreased cancer risk [10]. Therefore, depending on the extent of the infection and the host immune response, it is possible that *C. difficile* infection causes the release of various anti-inflammatory and tumoricidal cytokines that decrease the subsequent risk of PC. However, this association necessitates further evaluation.

Given the limited research on factors that reduce PC risk and the potential role of infections in having an anticancer effect, we sought to explore the relationship between *C. difficile* and PC. We hypothesized that *C. difficile* infection would be associated with a reduced incidence of PC.

## Materials and methods

A retrospective cohort study was performed and was exempt from institutional review board approval because all data were obtained from a database that provided de-identified patient information. A Humana Health Insurance Portability and Accountability (HIPAA)-compliant national database was provided by Holy Cross Health, Fort Lauderdale, Florida, for the sole function of academic research. The PearlDiver Mariner database was utilized in conjunction with the Bellwether interface to identify the patient population used in our study. PearlDiver contains over 41 billion HIPAA-compliant and de-identified patient records. The data within the database is derived from private insurance claims from Humana, United Healthcare, and Medicare. All payer types are included in the database, including self-pay, commercial, Medicare, and Medicaid. To meet the inclusion criteria, patients required active status in the database for at least eight years. Any PC patients diagnosed before *C. difficile* infection were not included in the study. PC diagnosis needed to have occurred after *C. difficile* infection for inclusion in this study.

The PearlDiver database was retrospectively reviewed with an inclusion window from January 2010 to December 2019, as these are the years available in the database utilized. The database was queried in May 2022. International Classification of Disease Ninth and Tenth Codes (ICD-9 and ICD-10), Current Procedural Terminology (CPT), and National Drug Codes were used to identify *C. difficile* infection and PC diagnosis. All types of PC and *C. difficile* infections were included in the initial search. Two groups of patients were identified within the database, which included patients with and without a history of *C. difficile* infection, respectively. Both groups were then propensity-matched by age range, sex, and Charlson Comorbidity Index (CCI) to minimize the effects of comorbidity-associated bias. The CCI score is used to predict the risk of death within one year of hospitalization for patients with specific comorbid conditions. The incidence of PC among both groups, with and without a history of *C. difficile*, was then assessed, and statistical analysis was performed using the PearlDiver statistical analysis software.

The groups were then matched again for antibiotic treatment exposure, including metronidazole, vancomycin, and fidaxomicin, to avoid the effects of treatment bias. Inclusion criteria in this part of the study were, therefore, expanded to require a history of exposure to the same treatment regimen, regardless of infection with *C. difficile*. There were various indications for exposure to the treatment criteria. The incidence of PC among both groups, with and without a history of *C. difficile*, was then again assessed, and statistical analysis was performed using the PearlDiver software.

PC incidence was the primary outcome measure of this study. Demographic breakdowns of patient age at diagnosis of PC and region of residence were subsequently assessed. Chi-square analyses, relative risk, and odds ratios (ORs) were utilized to analyze the results obtained from the database and assess the statistical significance of the correlations.

## Results

**Figure 1:**
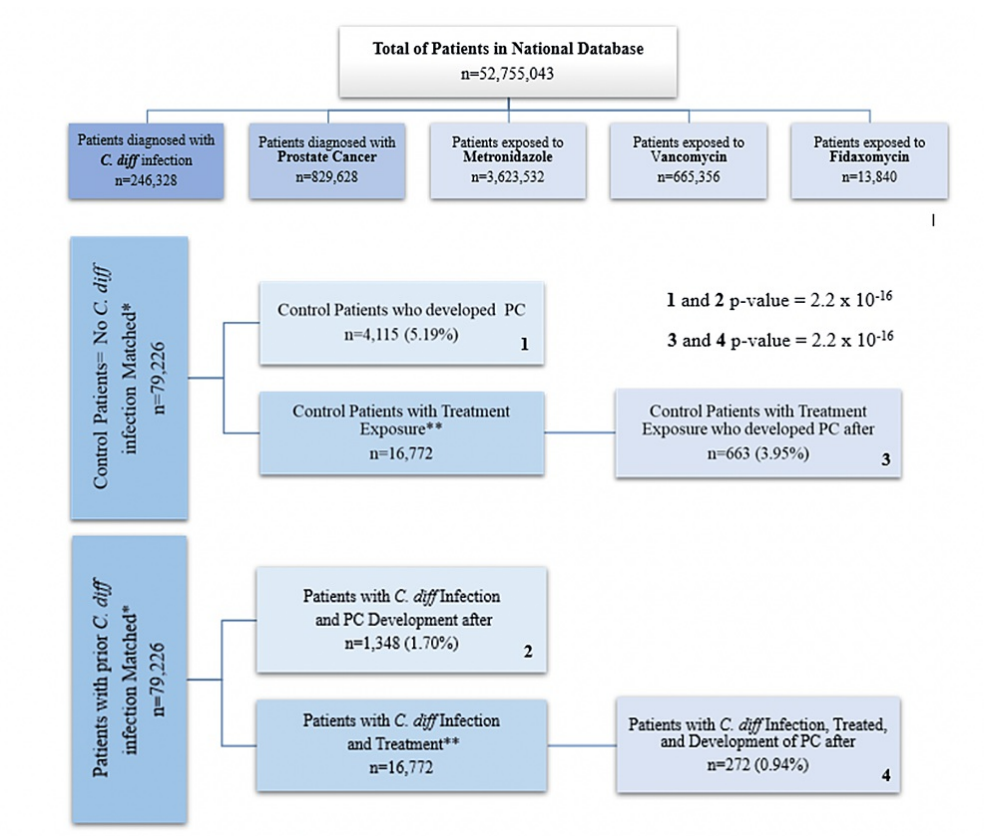
Stepwise description of the population contained at each level of the study with associated p-values for comparison groups. 1, 2: prior to treatment match; 3, 4: with treatment match; *: represents the populations that were matched by age range and CCI score; **: represents the groups treated with metronidazole and/or vancomycin and/or fidaxomicin. C. diff = *Clostridium difficile*; PC = prostate cancer; CCI = Charlson Comorbidity Index

Figure 1 demonstrates the stepwise results gathered at each step of our study (Figure 1). There were a total of 52,755,043 patients in the national database at the time of the query. The database was analyzed with an inclusion window from January 2010 and December 2019. There were 246,328 patients with a history of *C. difficile* infection in the database. There were 829,628 patients with a history of PC. The query resulted in 79,226 patients per group, matched for age range, sex, and CCI score. Subsequent matching for treatment exposure to metronidazole, vancomycin, and fidaxomicin resulted in 16,772 patients in both cohorts of patients, i.e., with and without *C. difficile* infection history. Patients taking metronidazole, vancomycin, and fidaxomicin were prescribed these medications for various indications. The most commonly used antibiotic among all groups was metronidazole, followed by vancomycin and fidaxomicin. The cohort without a history of *C. difficile* resulted in 663 (3.95%) patients with PC compared to 272 (0.94%) patients with PC in the cohort with a history of *C. difficile* infection.

The average time between treated *C. difficile* infection and PC diagnosis was 1,000 days. The average time between the control group with treatment exposure and PC diagnosis was 1,033 days.

**Figure 2:**
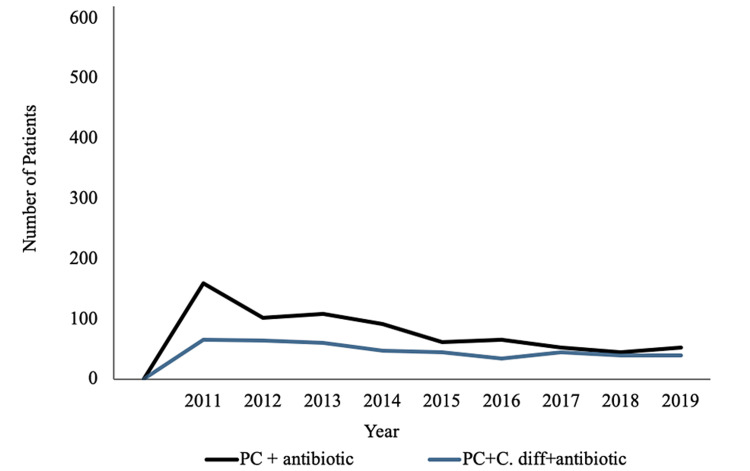
Incidence of PC compared by year of diagnosis. PC = prostate cancer; C. diff = *Clostridium difficile*

**Figure 3:**
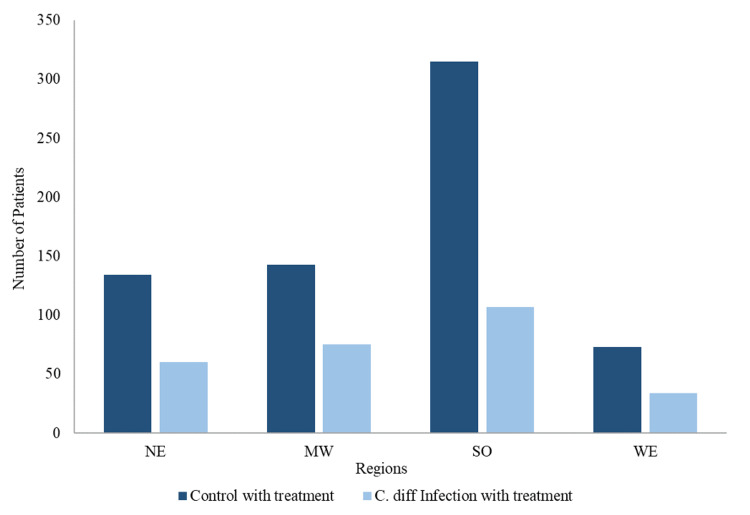
Regional distribution of PC patients. PC = prostate cancer; C. diff = *Clostridium difficile*; NE = northeast; MW = midwest; SO = south; WE = west

**Figure 4:**
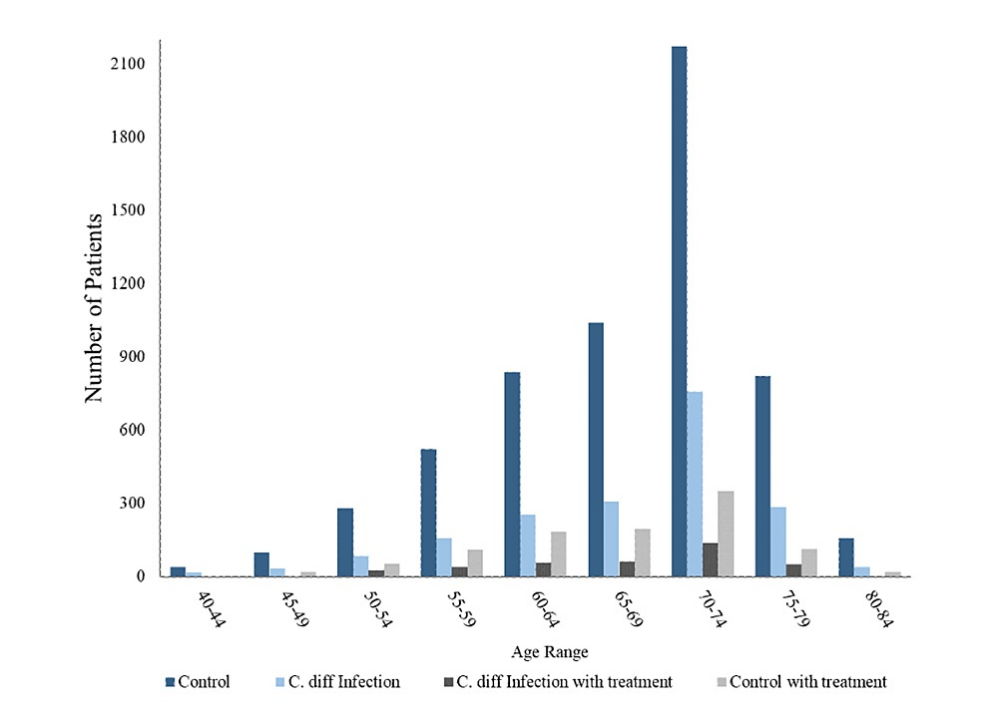
Age distribution of patients with PC. PC = prostate cancer; C. diff = *Clostridium difficile*

Figure 2 demonstrates an overall downward trend of PC diagnosis when compared by year of diagnosis regardless of prior *C. difficile* infection. Figure 3 displays the breakdown of the region of residence for patients diagnosed with PC included in this study (Figure 3). The southern region of the United States had the highest incidence of PC among all groups, while the western region exhibited the lowest incidence of PC among all groups (Figure 3). Figure 4 displays the incidence of PC diagnoses divided by age of diagnosis. The age of PC incidence peaked in patients aged 70-74 years among all groups (Figure 4).

The incidence of PC was 1.70% and present in 1,348 patients in the *C. difficile* group compared to 5.19% and present in 4,115 patients in the control group from a total of 79,226 patients per group prior to the treatment match (Figure 3). The decreased incidence in the *C. difficile* group was statistically significant with a p-value of 2.2 × 10^-16^, OR of 0.390, with a 95% confidence interval (CI) of 0.372-0.409, and a risk ratio of 0.409, with a 95% CI of 0.391-0.429 (Figure 3). The incidence of PC was 0.94% and present in 272 patients in the *C. difficile* group compared to 3.95% and present in 663 patients in the control group from a total of 16,772 patients per group with treatment match (Figure 3).

**Figure 5:**
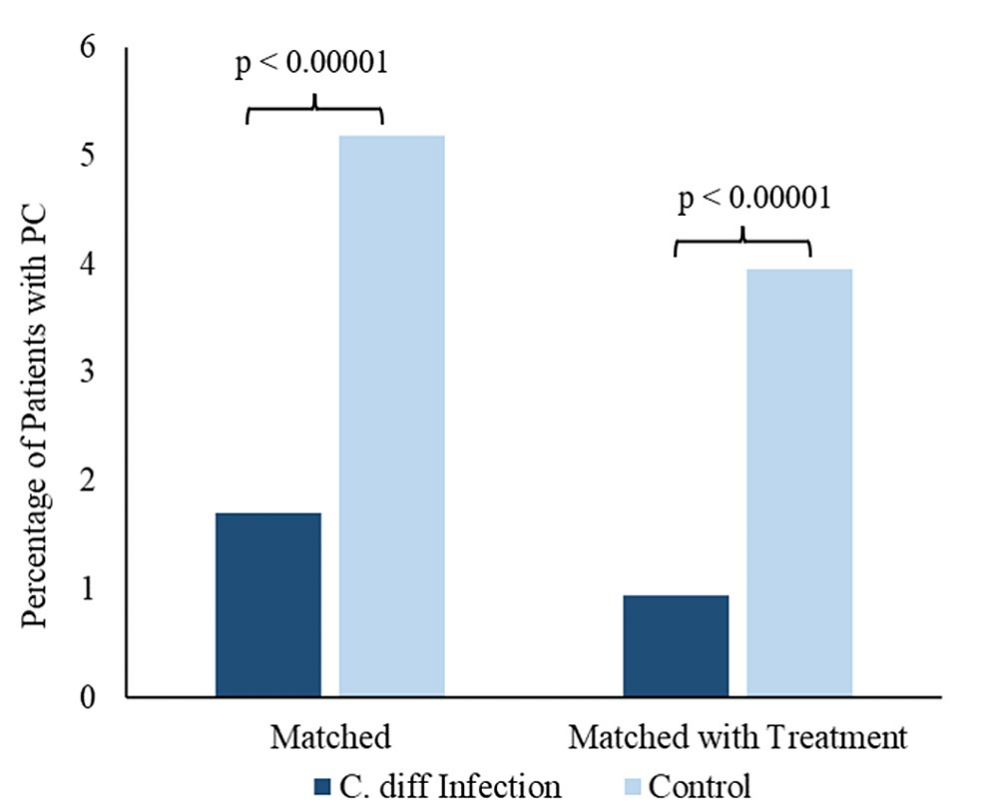
Incidence of PC is significantly decreased in patients with prior C. diff infection. C. diff = *Clostridium difficile*; PC = prostate cancer

Figure 5 demonstrates the percentage of patients with PC compared between patients with *C. difficile* infection history (dark blue bars) and without *C. difficile* infection (light blue bars). Figure 5 also shows that in comparisons of PC incidence among patients with and without *C. difficile* history, there is a decreased incidence of PC in patients with *C. difficile* history in analyses of both cohorts matched for age range, sex, and CCI only and matched for age range, sex, CCI, and treatment regimen (matched columns versus matched with treatment columns). The decreased incidence in the treated *C. difficile* group was statistically significant with a p-value of 2.2 × 10^-16^, OR of 0.467, with a 95% CI of 0.431-0.507, and a risk ratio of 0.481, with a 95% CI of 0.445-0.520 (Figure 5).

## Discussion

The results demonstrate that prior *C. difficile* infection may be a preventative factor for PC development. The data also revealed multiple demographic findings regarding *C. difficile* and PC. Although PC has been on the rise since 2010, our data support the overall decreased incidence of PC for the past two decades [11]. This may be attributable to decreased use of prostate-specific antigen (PSA) screening in efforts to oppose overdiagnosing PC that never reach symptomatic stages as well as improved lifestyle and possible environmental factors including infections. The incidence of *C. difficile* has been increasing in incidence despite preventative measures and remains one of the most common healthcare-associated infections [12]. This inverse trend in incidence between *C. difficile* and PC remains true among all analyzed groups. According to the Centers for Disease Control and Prevention, the rate of PC has shown to be the highest in the southern, northeastern, and midwestern states [13]. However, individual states such as Florida and Maine, which boast the highest percentage of residents above the age of 65 within the United States, experience lower PC incidence, demonstrating that PC risk extends beyond age and into environmental factors. One study showed that *C. difficile* infection had the highest incidence in the midwest followed by the southern and western regions of the United States [14]. Although the regional distribution in our study and prior studies do not take into account migration patterns and analyze different time periods, the data may not reflect the inverse association we expect based on our results.

The results of the study also demonstrate the average time between treatment exposure and PC diagnosis was approximately 2.7 years in the infected group and not much longer in the control group. These results reflect that there was enough time for prostate cells to experience detectable malignant transformation. PC doubling time among the cell lines PC-3 and DU-145 is 27.1 and 32.2 hours, respectively [15]. The doubling time has been shown to vary with the stage and grade of cancer, but mostly follows a slow linear growth rate with proportional PSA elevations [16,17]. These findings suggest that there was enough time between *C. difficile* diagnosis and PC diagnosis to reasonably attribute an effect.

The age distribution of our demographic results aligns with the well-known epidemiology of PC such that the incidence increases with age, most dramatically increasing above the age of 65 and peaking in the 70-74-year age group [13]. The rate of *C. difficile* infection also disproportionately affects older adults. Thus, the similar rates of increasing incidence in light of our study may show that the age of initial *C. difficile* infection may influence whether *C. difficile* takes on a more preventative role in PC. This trend potentially reflects the aging changes of the immune system along with all other relevant biochemical pathways in suppressing tumorigenesis. Furthermore, as most PC will remain in the asymptomatic stage as dysplastic lesions for several years to decades, environmental influence including infection along with the fixed non-modifiable risks may be responsible for progressing the tumor into a fully malignant symptomatic state [4].

Microbial dysbiosis disrupts the maintenance of metabolic homeostasis primarily by altering systemic immune responses [18]. The gut microbiota, in particular, has the ability to interact with distant organs which makes diseases such as distant cancers potentially subject to influence from dysbiosis arising from infections such as *C. difficile*. One study has shown that distinct microbiome signatures exist in healthy versus cancerous prostates which are further differentiated based on the grade and stage of cancer [18]. Although chronic inflammation hypothesized to stem from infection is commonly present in the adult prostate and considered a risk for carcinogenesis, our results demonstrate that single infectious microorganisms may each have unique roles.

With *C. difficile* infection, the potent glucosyltransferase toxins A and B have cytotoxic and proinflammatory properties that result in the upregulation of IL-8, IL-6, IL-1β, leukotrienes B4, and interferon-gamma [19]. These principal cytokines, specifically IL-8, initiate an inflammatory cascade with an excessive immune reaction leading to tissue destruction and dysbiosis [20]. Elevated IL-6 levels have been found in metastatic and castration-resistant PC compared to healthy patients, associated with shorter survival times, and progressive PC growth as a paracrine and autocrine growth factor for three different cell lines [21-23]. However, some studies have found a contradictory IL-6 role such that the cytokine exhibited a dose-dependent effect, inhibited androgen-dependent PC cell line growth, and had no effect on hormone-refractory PC cell lines [24,25]. Thus, the surge of IL-6 with *C. difficile* infection likely takes on the prohibitive side of its likely dual role in PC pathogenesis. Furthermore, dendritic cells detect *C. difficile* antigens such as surface layer proteins and cell wall proteins (Cwp66, Cwp84, CwpV) [8,26,27]. The response is an anti-inflammatory upregulation of cytokines IL-10, IL-23, and IL-4 [28]. Multiple studies have shown that anti-inflammatory drugs, diet, and response from commensal gut bacteria reduce chronic inflammation and related PC risk [8,29-32] However, the overall proinflammatory response also suggests the involvement of a different biologic pathway.

Another potential mechanism regarding the negative association between *C. difficile* infection and PC development involves the ability of *C. difficile* to alter androgen production. Within the same *Clostridium* genus, the commensal, *C. scindens*, was shown to increase steroid metabolites through the conversion of glucocorticoids into androgens, as well as dehydroxylation of primary bile acids into toxic secondary bile acids (deoxycholic acid, lithocholic acid (LCA)) [33,34]. Androgenic burden through point mutations, receptor amplification, and biosynthesis substantially underlies the development of PC [35]. High concentrations of secondary bile acids such as LCA are toxic endobiotics with tumor-promoting potential because of the consequent oxidative/nitrosative stress and genomic instability [36]. There are a limited number of commensals capable of producing these androgen and bile acid alterations, but with a prior *C. difficile* infection, the consequent dysbiosis may alter relative species abundance to favor microbes capable of enzyme modification of bile acids and steroids much less. While this potential mechanism is supported by our study and prior PC studies, it is purely hypothesis-based and necessitates further research.

There are several limitations of this study to be discussed. The design of the study, being an observational retrospective study, made adjusting for all confounding variables near impossible. The data were matched for age, sex, CCI, and treatment to minimize the effects of unknown confounders; however, this does not completely reduce the effects of these or other variables. The inability of our database to control for race, which is a known risk factor for PC, is a significant limitation that needs to be considered. We also did not have access to information regarding lifestyle factors, including consumption of red meat, which has been noted to play a role in PC prevention and development. Infection with *C. difficile*, while shown to reduce PC development via our study, is itself a dangerous infection with its own risks. Robust and further research is necessary before consideration of *C. difficile* for PC prevention or treatment.

## Conclusions

Despite multiple modifiable factors implicated in PC development, there have been limited established preventative and risk factors for PC. This study demonstrates a statistically significant novel association between *C. difficile* and reduced PC incidence. In light of this study, future research should be conducted to explore the potential role of tumoricidal and anti-inflammatory cytokines released by *C. difficile* in the prevention and treatment of PC.
